# Dysfunction of Cerebrovascular Endothelial Cells: Prelude to Vascular Dementia

**DOI:** 10.3389/fnagi.2018.00376

**Published:** 2018-11-16

**Authors:** Feixue Wang, Yu Cao, Lina Ma, Hui Pei, Wolf Dieter Rausch, Hao Li

**Affiliations:** ^1^Department of Geriatrics, Xiyuan Hospital of China Academy of Chinese Medical Sciences, Beijing, China; ^2^Department for Biomedical Sciences, Institute of Medical Biochemistry, University of Veterinary Medicine Vienna, Vienna, Austria

**Keywords:** vascular dementia, cerebrovascular endothelial cells, endothelial nitric oxide synthase, oxidative stress, inflammation, white matter lesion, synaptic plasticity

## Abstract

Vascular dementia (VaD) is the second most common type of dementia after Alzheimer’s disease (AD), characterized by progressive cognitive impairment, memory loss, and thinking or speech problems. VaD is usually caused by cerebrovascular disease, during which, cerebrovascular endothelial cells (CECs) are vulnerable. CEC dysfunction occurs before the onset of VaD and can eventually lead to dysregulation of cerebral blood flow and blood–brain barrier damage, followed by the activation of glia and inflammatory environment in the brain. White matter, neuronal axons, and synapses are compromised in this process, leading to cognitive impairment. The present review summarizes the mechanisms underlying CEC impairment during hypoperfusion and pathological role of CECs in VaD. Through the comprehensive examination and summarization, endothelial nitric oxide synthase (eNOS)/nitric oxide (NO) signaling pathway, Ras homolog gene family member A (RhoA) signaling pathway, and CEC-derived caveolin-1 (CAV-1) are proposed to serve as targets of new drugs for the treatment of VaD.

## Introduction

Vascular dementia (VaD) is an irreversible condition mainly attributable to various cerebrovascular diseases such as hypoperfusion, hypoxia, ischemia, and stroke, characterized by progressive cognitive decline, and difficulties with memory, language, and social ability, thereby imposing a huge burden on families, society, and the healthcare systems. Dementia is an age-related disease, and with the formation of an aging society in international community, the incidence of the disease has increased gradually. The World Health Organization (WHO) estimated that by 2050, the population with dementia would triple, up to >100 million. Of these cases, VaD will contribute about 15% (O’Brien and Thomas, 2015), rendering VaD as the second most common type of dementia after Alzheimer’s disease (AD).

International Classification of Diseases-10 (ICD-10) defines dementia that occurs after cerebrovascular disease as VaD. Most of VaD is caused by cerebral hypoperfusion (Mansour et al., 2018). Cerebrovascular endothelial cells (CECs) are usually the foremost in bearing the attack of hypoperfusion. CEC dysfunction not only causes blood–brain barrier (BBB) compromise, exposing neural cells to harmful substances (Di Marco et al., 2015), but also affects neurovascular coupling, making cerebral blood flow (CBF) cannot respond timely to neuronal activity (Hu et al., 2017). More importantly, cerebrovascular dysfunction and cerebral hypoperfusion affect mutually, turn into the two major contributors to VaD. However, the mechanism underlying the CEC impairment and its effect on cognition has not been completely elucidated. In this review, we elaborated on the structural and functional characteristics of CECs and summarized the potential mechanisms of CEC impairment in the early stage of hypoperfusion. Then, the pathological role of CECs in the neurological dysfunction and cognitive decline was exquisitely delineated in order to identify potential target molecules in the early stage of VaD; these can be regulated to prevent the onset of VaD or ameliorate the symptoms.

## Structural and Functional Characteristics of CECs

### CECs Are the Core Components of Blood–Brain Barrier (BBB)

#### Physical Barrier Between Blood and Brain

Blood–brain barrier is a barrier that effectively prevents harmful substances and large molecules from entering the brain. Structurally, CECs are the core components of BBB with some specific features. There are specific tight junctional proteins and a unique expression profile, but few fenestrae (Claudio et al., 1989; Iadecola, 2004) in CECs (Enerson and Drewes, 2006; Uchida et al., 2011; Kubo et al., 2015), which collectively maintaining strictly limited *trans*- and para-cellular transport cross BBB. The total length of capillaries in human brain was estimated about 400 miles with the surface about 20 m^2^ (Begley and Brightman, 2003), making CECs the crucial structure as a specific transport system and a chemical barrier.

The physical barrier property of BBB is primarily attributable to the junctional proteins between CECs that are classified into two types: tight junctions (TJs), and adherent junctions (AJs). TJs are mainly composed of claudins, occludins and junction adhesion molecules (JAMs); and vascular endothelial cadherin (VE-cadherin) is the main component of AJs. The TJs and AJs function together to seal the cleft between CECs and limit the hydrophilic molecules and blood cells entering the brain. The TJs seem to play the primary role in the sealing activity and the AJs are essential for the localization and stabilization of the TJs. Scaffold proteins including zonula occludens (ZO-1/2), cingulin, afadin (AF-6), and β-/γ-/p120-catenin (β-/γ-/p120-catenin) (Hawkins and Davis, 2005) are responsible for the anchoring of TJs and AJs to the cytoskeleton, and are indispensable in maintaining the structural integrity and functions of TJs and AJs. Alteration of the distribution or expression of these proteins can lead to increased paracellular permeability (Tornavaca et al., 2015).

#### Chemical Barrier—Transport System of CEC

Based on the differences in pathways, the substances cross the CECs via either paracellular pathway or transcellular pathway. The paracellular pathway of CECs mainly regulated by TJs opens only in disease state. Generally, the transcellular pathway can be classified into three categories, including (a) passive diffusion of lipophilic molecules, oxygen, and carbon dioxide; (b) facilitated diffusion of glucose, amino acids, and nucleosides via carriers/transporters; and (c) transport of macromolecules mediated by vesicles (Pardridge, 2005).

Some of specific expressed proteins act as the carriers/transporters mediating the transport of some essential nutrients or molecules into brain. The solute carrier superfamily (SLCs) is a kind of carrier proteins that are abundantly expressed in CECs and mediate the influx and efflux of small molecules such as glucose, amino acids, nucleotides, and peptides (Barar et al., 2016) cross BBB. Of these, the glucose transporter-1 (GLUT-1) is vital in maintaining the normal brain function as the brain has little energy reserve. GLUT-1 has to function normally to ensure the sufficient glucose for neurons. Amino acids are essential for neurotransmitter and protein synthesis in the brain. As an excitatory neurotransmitter, glutamate can be maintained at a low extracellular level in the brain by normal function of excitatory amino acid transporters (EAATs) expressed on the abluminal side of CECs (O’Kane et al., 1999; Hawkins, 2009; Barar et al., 2016), preventing neurons from being overstimulated. In some conditions, the alteration of expressions of GLUT-1 and EAATs in CECs could lead to cognitive impairment (Zhang et al., 2013a).

Some lipids, cholesterol, and long-chain fatty acids may enter the brain tissue facilitated by the ATP-binding cassette (ABC) transporter family. The ABC family is primarily located on the luminal side of CECs (Theodoulou and Kerr, 2015). Of these, *ABCB1*-encoded *P*-glycoprotein (*P*-gp) is essential for the removal of the metabolizing toxins, foreign substances, or drugs from the brain (De Lange et al., 2018). If the ABC transporters malfunction, toxins would accumulate in the brain and advance the occurrence or progression of neurological disease (Chan et al., 2011).

Transport mediated by vesicles also plays a vital role in maintaining the brain homeostasis even though there are fewer vesicles in CECs than that in peripheral endothelium (Reese and Karnovsky, 1967). Transcytosis is the foremost way for the macromolecules, such as insulin and transferrin, entering the brain (Jiang et al., 2017). Macromolecules bind to receptors on CEC membrane and are transported by vesicles (such as caveolae and exosomes). These vesicles are proposed to serve as targets in treating central nervous system (CNS) diseases. Morphological evidence has shown that the caveolae may be the main form of vesicles of CECs (Simionescu et al., 2009). Caveolae are specialized cystic structures, occupying up to 50% of the plasma membrane surface (Gratton et al., 2004). The marker proteins of caveolae are caveolin 1–3 (CAV-1, 2, 3), which are abundantly expressed in CECs and involved in signal transduction, vesicular transport, and BBB permeability (Barar et al., 2016). First, caveolae are involved in the *trans*-membrane transport of a variety of macromolecules such as albumin and lipoproteins (Xu L. et al., 2015). Moreover, caveolin has a large amount of microdomains that bind to multiple signaling molecules, such as G protein-coupled receptors (GPCRs) (such as acetylcholine, bradykinin, and endothelin), ion channels (such as Ca^2+^-ATPase), platelet-derived growth factor receptor (PDGFR), and transforming growth factor-β (TGF-β) receptors (Dessy et al., 2010), involved in signal transduction. Secondly, in resting state, CAV-1 bind to endothelial nitric oxide synthase (eNOS) to keep it inactivate (Gratton et al., 2000). Whenever needed, eNOS can be dissociated from CAV-1 to promote the production of the nitric oxide (NO) (Sharma et al., 2011). Importantly, caveolae can also regulate BBB permeability by affecting CEC transcellular transport. It has been shown that under ischemic conditions, caveolae can mediate high permeability of BBB (Chen F.Q. et al., 2016; Nahirney et al., 2016).

### CEC Are the Key Component of NVU

The interaction between neurons, glial cells, and cerebral blood vessels [including CEC, vascular smooth muscle cells (VSMCs), and pericytes] forms a dynamic functional unit known as NVU. The signal communication between the components of NVU not only ensure the fine regulation of CBF to fulfill the metabolic needs, but also play a major role in brain development, nourishment and repair, and BBB functioning (Andreone et al., 2015; Iadecola, 2017). The close association of CECs and neural cells allows the signals by CEC damage to be transduced to the brain [e.g., the impaired NO function alters the synaptic plasticity (Blackshaw et al., 2003)]. Therefore, signal disturbances of NVU might underlie the nerve injury, apoptosis and neurological dysfunction (Lourenco et al., 2018; Maiuolo et al., 2018). Thus, CECs are not only the primary components of BBB structurally but also the key link of NVU activities functionally.

#### eNOS/NO Signaling Pathways in CEC

In CNS, NO not only is the most important endothelium-derived vasodilation factor involved in the regulation of baseline vascular tone and functional hyperemia, but also plays a critical role in the maintenance of neurological functions. Thus, it has been regarded as a crucial index in judging whether or not the vascular function is normal.

Nitric oxide is produced from L-arginine, and the reaction is catalyzed by eNOS, which can be activated in a calcium-dependent manner (Vanhoutte et al., 2016). At rest, eNOS localizes in caveolae. CAV-1 binds to its calcium-binding domain and prevents calcium from activating it, leaving eNOS inactive (Alderton et al., 2001; Dessy et al., 2010). Under the stimuli such as vascular endothelial growth factor (VEGF) and shear stress, the increased intracellular Ca^2+^ and the calmodulin/Ca^2+^ (CaM/Ca^2+^) promotes the dissociation of eNOS from CAV-1 (Dessy et al., 2010). Then, eNOS is transferred into the cytoplasm, where it couples with the co-factors such as tetrahydrobiopterin (BH4) to form a couplet that can produce NO (Fleming, 2010; Michel and Vanhoutte, 2010; Forstermann and Sessa, 2012; Zhao et al., 2015). In this process, heat shock protein 90 (HSP90) binding to eNOS not only promotes the phosphorylation (activation) of eNOS but also enhances its dissociation from CAV-1 (Garcia-Cardena et al., 1998; Gratton et al., 2000; Dessy et al., 2010; Michel and Vanhoutte, 2010; Natarajan et al., 2015; Vanhoutte et al., 2016). eNOS can also be phosphorylated through signaling pathways of phosphoinositol 3 kinase (PI3K)/protein kinase B (PKB/Akt), cyclic adenosine monophosphate/protein kinase A (cAMP/PKA) and extracellular regulated protein kinases 1/2 (ERK1/2)/mitogen-activated protein kinase (MAPK) and stimulate the release of NO (Michel and Vanhoutte, 2010; Forstermann and Sessa, 2012; Heiss and Dirsch, 2014; Vanhoutte et al., 2016).

After production from CECs, NO interacts with soluble guanylate cyclase (sGC) in VSMCs to generate cyclic guanosine monophosphate (cGMP) and to transduce signals via cGMP/protein kinase G (PKG) pathway, thereby participating in downstream biological effects (Vanhoutte et al., 2016). Normally, on one hand, NO can inhibit platelet aggregation and maintain the low expression of adhesion molecules on the surface of endothelial cells and inhibit the adhesion and penetration of leukocytes (Gao, 2010; Vanhoutte et al., 2017). On the other hand, NO can activate the peroxisome proliferator-activated receptor-γ (PPARγ) via the NO/cGMP/PKG signaling pathway and promote the mitochondrial DNA and protein synthesis, involved in regulating mitochondrial function and oxidative stress (Nisoli et al., 2004; Miller et al., 2013). In this concept, NO is a protective factor against inflammation and oxidative stress in both systemic and central vasculature.

In CNS, CEC-derived NO is a vital mediator to maintain the vascular tone and regulate CBF (Atochin and Huang, 2010). This function is demonstrated in mammal experiments (Chillon and Baumbach, 2004; Wilkerson et al., 2005). NO derived from neuronal NOS (nNOS) seems play the major role in functional hyperemia (Attwell et al., 2010), while endothelium-derived NO is regarded to be a vital player in CBF auto-regulation. In response to shear stress and other stimuli, such as acetylcholine (Ach), bradykinin (Arnal et al., 1999), eNOS was activated and NO released from CECs interacts with sGC in VSMCs to relax them and increase regional blood flow. Furthermore, besides the role in CBF regulation, eNOS/NO also regulates neurogenesis, axonal outgrowth and synaptic plasticity. CEC-derived eNOS/NO signaling pathway has been found to underlie the maintenance of synaptic plasticity in hippocampus and cortex (Haul et al., 1999; Blackshaw et al., 2003; Doreulee et al., 2003), participating in basic memory formation (Katusic and Austin, 2014). CEC-derived NO can act on GC-coupled NO receptors in axons, causing increased cGMP expression, which further depolarizes the cell membrane and promotes the propagation of the action potential (Garthwaite et al., 2006).

In CNS, with the catalysis of eNOS and nNOS, low level of NO is produced to exert a protective effect on the blood vessels and neural cells. However, during inflammation, inducible NOS (iNOS) is activated to produce a level of NO much higher than that in the normal condition (Dorheim et al., 1994; Alderton et al., 2001). The excess NO causes neurotoxicity, impairs mitochondrial function, and induces apoptosis as a free radical (Brown, 2010).

#### Neurovascular Coupling (NVC)

The process of NVC is the foundation of functional hyperemia. Pericytes enwrapping capillaries and VSMCs around arterioles are the two effector cell types in response to the signals transmitted from neurons or astrocytes (Hall et al., 2014), though the debate on the contracting property of pericytes remains (Hill et al., 2015; Cudmore et al., 2017). Activated neurons or interneurons could release signals directly or indirectly acting on astrocytes, endothelium, VSMCs or pericytes to dilate arterioles or capillaries, initiating the CBF increase. In NVC, one predominant mediator to relax VSMCs is NO derived from nNOS produced by the postsynaptic neurons (Attwell et al., 2010). Although endothelial NO is endowed with the same function (as mentioned in the last part), to what extent it works in NVC still needs to be investigated. Astrocytes play an instrumental role in NVC, which has been studied a lot because of their intimate contact with both neurons and vessels, but the contradictions remains (Iadecola, 2017). Astrocytes are generally acknowledged to be activated by the glutamate released by activated neurons and to release a series of arachidonic acid (AA) metabolites that act on the VSMCs to dilate the vessels (Mishra, 2017). Another proved role of astrocytes in NVC is their effect to dilate capillaries (Longden et al., 2017). The overspill of K^+^ from astrocytes activates the inward-rectifier K^+^ (*K*_IR_) channels in CECs, which are hyperpolarized and propagate the signal retrogradely to the upstream feeding arterioles (Longden et al., 2017), avoiding the phenomenon of “flow steal.” It is proposed that the signaling from astrocytes to VSMCs is propagated by CECs but not from astrocyte to VSMCs directly. Capillaries in the brain were estimated to be responsible for 84% of CBF increase induced by neural activity (Hall et al., 2014), showing the crucial role of CECs in regulating CBF.

#### Neurovascular Trophic Coupling (NVTC)

In addition to regulation of CBF, CECs can also express multiple nutrient factors and growth factors that provide nourishment and support to neural cells, which is regarded as NVTC. In brain development, with expression of VEGF, brain-derived neurotrophic factor (BDNF) or basic fibroblast growth factors (bFGF), CECs can promote the differentiation of neural stem cells (NSCs) into different kinds of neural cells. And under injury, CECs support the survival of existing neurons and promote the repair and regeneration of neurons and synapses, whereby CECs play a positive role in memory and cognitive function.

## CEC Dysfunction Caused by Cerebral Hypoperfusion — a Prelude to VaD

Vascular dementia is mainly caused by cerebrovascular disease. Aging, obesity, hypertension, diabetes, hypercholesterolemia, hyperhomocysteinemia, atherosclerosis, and stroke are the risk factors for VaD (Roman, 2003; Lu F.P. et al., 2009; Maron and Loscalzo, 2009; Allan et al., 2011; Gottesman et al., 2014; Yang T. et al., 2017). Due to these risk factors, cerebral hypoperfusion occurs, which has been shown to cause attention and memory deficits as well as cognitive impairment (Duschek et al., 2005; de la Torre, 2012; Kitamura et al., 2012). Chronic or acute cerebral hypoperfusion leads to oxidative stress and activation of CECs, which is the fundamental pathology of CEC dysfunction and the subsequent brain lesions. Owing to the critical functions and wide-ranging effects of CECs, CEC dysfunction caused by cerebral hypoperfusion may be a key initiation event for VaD (Faraci, 2011; De Silva and Faraci, 2016; Hu et al., 2017). Patients with VaD often appear with focal changes in CECs, including decreased mitochondrial content (de la Torre and Aliev, 2005), increased pinocytosis, loss of TJs, disruption of BBB, and decreased capillary density (Brown and Thore, 2011; Pires et al., 2013). These changes might have existed before the onset of VaD. If measures can be taken to improve CEC function before the occurrence of dementia symptoms, the development of VaD can be slowed or prevented. This could be adapted as a potential approach to treat VaD.

### Basic Pathological Changes of CECs

#### Oxidative Stress

Increased production of reactive oxygen species (ROS) or reactive nitrogen species (RNS) in the cerebral vasculature (Strasser et al., 1997) leads to oxidative stress, which is a major cause of CEC dysfunction and is pivotal in the development of cerebral vascular diseases.

In the vasculature, nicotinamide adenine dinucleotide phosphate (NADPH) oxidase, mitochondria and cyclooxygenase (COX) are the top three sources of ROS (Lee and Griendling, 2008). The large amount of mitochondria are the major sources of ROS in CECs (Acuna-Castroviejo et al., 2001). ROS can cause the dysfunction of anti-oxidants such as glutathione peroxidase (GPx) and superoxide dismutase (SOD) in mitochondria, leading to progressive and on-going oxidative stress of CECs.

Nicotinamide adenine dinucleotide phosphate oxidase is also a key source of ROS in CECs (Richter et al., 1988) that has been investigated thoroughly. In aging, obesity, hypertension, atherosclerosis, stroke, and AD models, the expression of NADPH oxidase 2 (NOX-2), a subunit of NADPH oxidase, is significantly upregulated. Antioxidants or blockade of NOX-2 can result in the improvement of CEC and NVC function (Erdos et al., 2006; Park et al., 2007, 2017; Judkins et al., 2010; Ma et al., 2017; Qin et al., 2017). The upregulation of NOX-2 in aged CECs is primarily attributed to the significant increase in the level of tumor necrosis factor-α (TNF-α) during aging (Ungvari et al., 2003). In hypertension, CECs are extremely sensitive to Angiotensin II (ANG II), which binds to ANG II receptor type-1 (AT-1) and significantly increases the NOX-2 activity, thereby promoting ROS production (Li W.J. et al., 2016). The inhibition of AT1 can reduce the production of ROS. Hydrogen peroxide (H_2_O_2_) is more stable than superoxide anion; it can freely penetrate the cell membrane and activate the NADPH oxidase in CECs to further produce the superoxide substances, expanding the oxidative stress (Faraci, 2006b). Interestingly, *NOX-2* knockout mice showed impaired synaptic plasticity and cognition (Kishida et al., 2006). Moreover, NOX-2-derived ROS might aid in maintaining CBF and neural function. Thus, the strategy of taking NADPH oxidase as a therapeutic target to treat cerebrovascular diseases requires careful consideration.

Another noteworthy source of ROS is COX-2 that releases only a small amount of ROS during catalysis of AA to prostaglandin E2 (PGE2) (Marnett et al., 1999), and its oxidative capacity cannot be ignored owing to the increase in COX-2 activity in the elderly (Chung et al., 2006). Interestingly, ROS is a contributor to the over-expression of COX-2 under pathological state (Perez-Giron et al., 2014). Moreover, COX-2 also has the pro-inflammatory property. Interaction of ROS and COX-2 forms a vicious circle of oxidative stress and inflammation.

Cerebrovascular endothelial cells encompass a variety of molecules and signaling pathways that are resistant to oxidative stress. Nuclear factor E2-related factor-2 (Nrf-2) is a transcription factor that is sensitive to oxidative stress. Under oxidative stress, it can induce the expression of multiple antioxidant enzymes such as SOD and glutathione (GSH) (Song et al., 2014). PPARγ is a nuclear receptor central to energy balance and inflammatory processes. It acts as a key link in the pathways of Nrf-2, Wnt/β-catenin, and forkhead box factor of the O class 1 (FoxO1), exerting an anti-oxidative stress role (Polvani et al., 2012). A previous study has shown that PPARγ pathway protects the endothelial function in oxidative stress (Beyer et al., 2008). Silent information regulatory factor-1 (SIRT-1) can be activated in oxidative stress; it can inhibit NADPH oxidase (Zarzuelo et al., 2013) and promote the expression of manganese-dependent SOD (MnSOD), catalase (CAT) and other genes that reduce the oxidative stress in AD, atherosclerosis, ischemia-reperfusion injury, diabetes, and aging (Chong et al., 2012; Kitada et al., 2016). FoxO is a member of the forkhead transcription factor subfamily and plays a crucial role in the process of oxidative stress. FoxO signaling not only reduces the mitochondrial ROS production but also induces the expression of MnSOD and CAT (de Keizer et al., 2011). Recently, FoxO has been shown to exert a protective role in cognitive function (Maiese, 2016, 2017). Still, the controversial opinions exist. There are studies showing FoxO could induce ROS production, and exert a negative role in brain function (Manolopoulos et al., 2010; Li J. et al., 2016; Sun et al., 2018). Therefore, a balance of FoxO level should be deliberated if it is considered to be a therapeutic target.

#### CEC Activation

Cerebrovascular endothelial cell activation is considered to be the key initial step in the process of cerebral senescence, and also is one of the underlying pathological changes of VaD risk factors. Under the effect of altered intraluminal shear stress, VEGF or ROS, there is an increase in the calcium concentration in CECs, which is an early cause of endothelial activation. This phenomenon promotes the CECs to express P-selectin and chemokine, enhancing the recruitment of leukocytes and platelets (Dole et al., 2005; Jin et al., 2010). Activated leukocytes and platelets release TNF-α and interleukin-1 (IL-1), which further mediate CEC activation and stimulate expression of vascular cell adhesion molecule-1 (VCAM-1) and intercellular adhesion molecule-1 (ICAM-1) rapidly (Diamond et al., 1991; Rivera-Nieves et al., 2008), promoting the firm adhesion and migration of leukocytes.

In addition, innate immune system plays a role in CEC activation. In the condition of oxidative stress, as well as in aging, hyperlipidemia, atherosclerosis, and stroke, Toll-like receptor-2/3/4/6 (TLR-2/3/4/6) are up-regulated or activated (Salminen et al., 2008; Nagyoszi et al., 2010; Zhang et al., 2012). Especially, TLR-4 has been shown to activate ICAM-1 and contributes to the inflammatory phenotype of CECs (Lee K.M. et al., 2015; Seok et al., 2015). The TLR-4/myeloid differential protein-88 (MyD-88)/nuclear factor-κB (NF-κB) pathway is central in the regulation of CEC activation (Wilhelm et al., 2017). By interaction with endogenous ligands of TLR-4, e.g., oxidized low-density lipoprotein (oxLDL) by ROS (Yang et al., 2005), high mobility group box protein 1 (HMGB1) (Chen et al., 2015) or fibrin (Zhang et al., 2012), TLR-4/MyD-88/NF-κB pathway was activated, with a series of pro-inflammatory factors released and NF-κB activated, while activation of NF-κB signaling pathway plays a major role in the inflammatory response of CECs. In elderly and hypertensive patients, ROS and TNF-α levels are elevated, both of which can activate inhibitor of κB (IκB) kinase (IKK) and promote the activation of NF-κB (Chen and Goeddel, 2002; Nakajima and Kitamura, 2013). The major targets of NF-κB are genes of IL-6, IL-1β, Monocyte chemoattractant protein-1 (MCP-1), COX-2, p53, MnSOD, and NADPH oxidase (Pasparakis, 2009). It not only has a strong pro-inflammatory effect but also promotes the release of ROS and RNS, exacerbating the oxidative stress of CECs (El Assar et al., 2013).

Multiple anti-inflammatory pathways are harbored in the human body. IL-10 is a potent inhibitor of NF-κB. It can degrade NF-κB, affect its binding to deoxyribonucleic acid (DNA) and reduce the associated inflammatory response. In atherosclerotic and diabetic disease models, endogenous IL-10 expression was found to be reduced, resulting in vascular dysfunction (Heldt et al., 1999; Gunnett et al., 2000, 2002; Caligiuri et al., 2003). The PPARγ and SIRT1 already described above not only resist the oxidative stress but also exert anti-inflammatory effect; enhancing the activity of PPARγ and SIRT1 can reduce the inflammatory response of CECs and improve the neural function (Gano et al., 2014). In addition, the physiological level of NO released by CECs exerts anti-inflammatory effect by activating adenosine 5′-monophosphate-activated protein kinase (AMPK) (Zhang et al., 2008), and also directly blocks the NF-κB signaling pathway, thereby decreasing the expression of inflammatory factors (Grumbach et al., 2005). Thus, the low-concentration of NO plays an indispensable role in maintaining normal vascular function.

#### Impairment of eNOS/NO Signaling Pathway

Activation and oxidative stress of CECs result in the impairment of eNOS/NO signaling pathway, which represents a significant indicator of CEC dysfunction (Forstermann, 2010). Oxidative stress plays a major role in the NO signaling impairment, which primarily manifests in the following aspects:

First, the production of NO is decreased. eNOS activity is the decisive factor affecting NO production. ROS can decrease eNOS activity through the Ras homolog gene family member A(RhoA) pathway (Forstermann and Munzel, 2006). Since dimethylarginine dimethylaminohydrolase (DDAH) is sensitive to ROS, it could be blunted by ROS, thus cannot degrade asymmetric dimethylarginine (ADMA) (Lin et al., 2002; Scalera et al., 2004), which is an endogenous inhibitor of eNOS (Vallance and Leiper, 2004; Boger, 2006; Sasser et al., 2014). Significantly elevated ADMA can be found in the elderly, hypercholesterolemia, and atherosclerosis (Boger et al., 1998; Antoniades et al., 2011). Importantly, in patients with diabetes and hypertension, excess ROS was found to oxidize tetrahydrobiopterin (BH4) to BH2, resulting in uncoupling of eNOS and BH4 (Vanhoutte et al., 2016). The uncoupled eNOS interacts with oxygen (O_2_) to produce superoxide instead of catalyzing L-arginine to produce NO, thus forming a vicious circle of oxidative stress. Restoring BH4 level can alleviate endothelial dysfunction (Zheng et al., 2003). As a consequence, uncoupled eNOS has also been considered as one of the sources of ROS in vascular endothelium (Lee and Griendling, 2008). Elevated arginase by H_2_O_2_ hydrolyzes L-arginine, the substrate for NO, also contributing to the reduced production of NO (Faraci, 2006a).

Second, ROS leads to a decrease in NO bioavailability. Increased O^2-^ reacts with NO to form peroxynitrite (OONO^-^), resulting in a decrease of NO, which is the main mechanism underlying ROS-mediated endothelial dysfunction. OONO^-^ is a type of RNS which is also one of the main substances that cause oxidative stress. First, RNS can oxidize sGC and reduce its reactivity to NO, leading to further loss of NO signaling (Munzel et al., 2005; Stasch et al., 2006). OONO^-^ can promote uncoupling of eNOS and BH4 (Zou et al., 2002), decrease the activity of MnSOD (Faraci and Didion, 2004) and glutathione reductase, and increase the expression of iNOS (Pacher et al., 2007), leading to extensive oxidative stress. With the action of iNOS, NO is far beyond the physiological requirements. High level of NO is also a type of RNS that causes damage to CECs.

Given the widespread role of NO, damaged eNOS/NO signaling pathways may exert severe effects on CNS. First, reduced NO production or decreased bioavailability leads to impaired CBF regulation, leading to further reduction in cerebral perfusion. Secondly, reduced NO is also a major contributor to vascular inflammation. The attenuated function in inhibiting platelet aggregation and leukocyte adhesion of lower NO not only promotes the CEC activation but also leads to the penetration of inflammatory cells and toxic proteins into the brain via transcellular and intercellular pathways, conducting neurotoxic effects (De Caterina et al., 1995). Third, the eNOS/NO signaling pathway is critical for maintaining the memory and learning ability. Since eNOS/NO signaling underlies BDNF production in CECs (Monnier et al., 2017; Pedard et al., 2017), the damage to eNOS/NO can hinder the synthesis of BDNF, thereby weakening synaptic plasticity and neural regeneration (Doreulee et al., 2003), and this may be one of the basic causes of early damage to synaptic plasticity.

**FIGURE 1:**
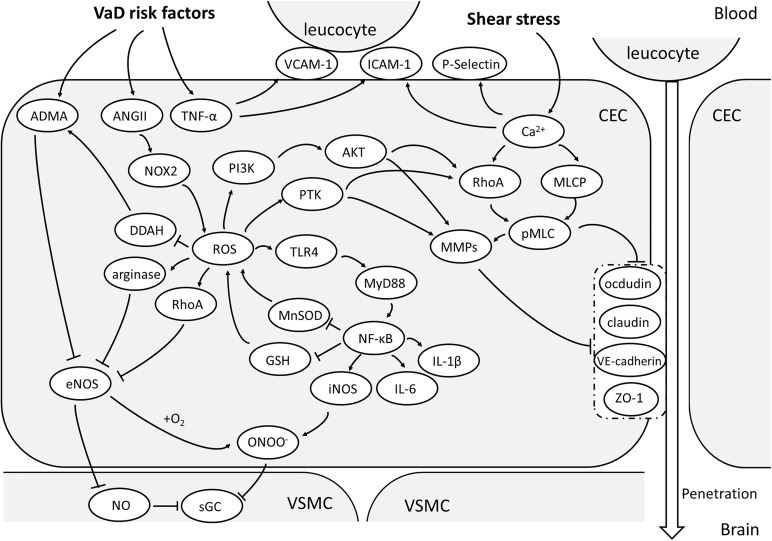
Oxidative stress and activation of CEC. With the vascular risk factors, NOX-2 is the main source of ROS in CECs. With the effect of ROS, ADMA will accumulate and arginase activated, both of which will reduce NO production and increase OONO^-^ via inhibiting eNOS. Together with a large amount of NO due to activation of TLR/MyD88/NF-κB pathway, OONO^-^ can be produced and attenuate the sensitivity of sGC, making the malfunctioning of VSMC vasodilation. NF-κB pathway is an important upstream mechanism of the expression of pro-inflammatory factors. ROS could also enhance MMP expression and activate RhoA/pMLC pathway through PTK or PI3K/Akt signaling and consequentially relocate or degrade TJs and AJs, resulting to high paracellular permeability of CECs. The elevated TNF-α promotes the expression of ICAM-1,VCAM-1 and P-selectin, which helps in leucocyte adhesion to CECs and penetration into brain. (AJs, adherent junctions; AMDA, asymmetric dimethylarginine; AKT, protein kinase B; ANGII, angiotensin II; CEC: cerebrovascular endothelial cell; DDAH, dimethylarginine dimethylaminohydrolase; eNOS, endothelial nitric oxide synthase; GSH, glutathione; ICAM-1, intercellular adhesion molecule-1; iNOS, inducible nitric oxide synthase; MLCP, myosin light chain phosphorylatase; MMPs, metalloproteins; MnSOD, manganese-dependent superoxide dismutase; MyD88, myeloid differential protein-88; NF-κB, nuclear factor-κB; NO, nitric oxide; NOX-2, nicotinamide adenine dinucleotide phosphate oxidase 2; OONO^-^, peroxynitrite; PI3K, phosphoinositol 3 kinase; pMLC, phosphorylated myosin light chain; PTK, protein tyrosine kinase; RhoA, Ras homolog gene family member A; ROS, reactive oxygen species; sGC, soluble guanylate cyclase; TJs, tight junctions; TLR4, Toll-like receptor 4; TNF-α, tumor necrosis factor-α; VCAM-1, vascular cell adhesion molecule-1; VE-cadherin, vascular endothelial cadherin; VSMC, vascular smooth muscle cell; ZO-1, zonula occludens 1).

The two pathological processes, oxidative stress and activation/inflammation, impair eNOS/NO signaling in CECs via a series of overlapped mechanisms (Figure 1), finally leading to CEC malfunctioning in maintaining the BBB function and regulating CBF.

### Functional Impairment of CECs

#### BBB Dysfunction

Blood–brain barrier dysfunction is a very fundamental pathology that causes VaD (Di Napoli and Shah, 2011). It has been shown that the level of BBB damage in the hippocampus could predict the degree of cognitive impairment in elderly patients (Montagne et al., 2015). With the risk factors such as aging and hypertension, which always coexist with cerebral hypoperfusion, CECs are prone to be compromised.

##### Increased paracellular permeability of CEC

Tight junction and AJ dysfunction is responsible for the increased paracellular permeability to hydrophilic molecules or inflammatory cells of CECs, which has been demonstrated both *in vitro* and *in vivo*. TJ dysfunction has been studied thoroughly. Some mechanisms underlying TJ dysfunction in cerebral hypoxia, ischemia or oxidative stress have been proven. Among these, the phosphorylated myosin light-chain (pMLC) is central to mediate TJ redistribution and BBB dysfunction. In a BBB model of bEnd3 cell line, an increase in pMLC is detected by proteomics within 1 h of hypoxia, followed by the redistribution of ZO-1 and BBB leakage, which could be prevented by blockade of MLC phosphorylation (Hicks et al., 2010), indicating the pMLC is a determinant link in this process (Haorah et al., 2005; Shi et al., 2016; Izawa et al., 2018). Actually, phosphorylation of MLC also underlies AJ impairment, which is also implicated in increased paracellular permeability (Haidari et al., 2011, 2012). One possible pathway leading to TJ and AJ relocation is that the increased concentration of cytosolic Ca^2+^ activates MLC kinase (MLCK), increasing the phosphorylation of MLC and the subsequent contraction of actin (Wang et al., 2012). Consequently, occludin, VE-cadherin, β-catenin and ZO-1, attached to actin, redistribute and lose their original sealing function, leading to BBB leakage (Brown and Davis, 2002). In-between the signaling transduction from elevated Ca^2+^ to MLC phosphorylation, the RhoA/ROCK has been revealed to be a major mediator (He et al., 2011; Izawa et al., 2018). In the model of BBB disruption induced by hemoglobin, the levels of RhoA and ROCK2 were significantly upregulated, concomitant with enhanced pMLC and diminished claudin-5 in endothelial cells, as well as increased matrix metalloproteinase-9 (MMP-9) (Fu et al., 2014). It also indicates that MMP-9 may participate in the mechanism of TJ dysfunction, while oxidative stress has been shown to play a major role in upregulation of MMPs or downregulation of tissue inhibitor of metalloproteinase -1/2 (TIMP-1/2) in a protein tyrosine kinase (PTK)-dependent manner or via PI3K/Akt pathway (Haorah et al., 2007; Schreibelt et al., 2007). *SOD1/2* knockout mice express excessive proMMP-9 and MMP-9 after cerebral ischemia with severe BBB damage detected by Evans blue extravasation (Gasche et al., 2001; Maier et al., 2004), which is also validated by other studies (Yang et al., 2007; Zhang et al., 2018).

We discussed the positive effect of eNOS on CBF regulation and neurological functions, however, there are also studies indicating that the eNOS is an important mediator of TJ dysfunction and the consequent BBB leakage, while knock-down or inhibition of eNOS can abrogate this adverse effect (Beauchesne et al., 2009; Argaw et al., 2012; Ko et al., 2015). These studies did not simultaneously investigate the impact of eNOS inhibition on CBF regulation, so the conclusion that the role of eNOS is beneficial or detrimental in CNS pathology should not be reached indiscreetly unless much more studies are conducted on it.

##### Increased transcellular transport of CECs

Increased transport through transcellular pathways of CECs is another cause of impaired BBB. Some studies demonstrate that early in the stroke, transcytotic vesicles appear to increase before the alteration of TJs function (Krueger et al., 2013; Haley and Lawrence, 2017). Increased transcellular transport is closely related to the increase of caveolae in CECs. Additionally, the occurrence of cerebral ischemia or neuroinflammation is always accompanied by the increase in caveolae (Nahirney et al., 2016) together with BBB disruption. After cerebral hemorrhage, *CAV-1* knockout mice exhibit reduced leukocyte adhesion and MMP-9 expression (Chang et al., 2011). Caveolae can mediate the entry of various cytokines and toxic proteins into the brain. For instance, caveolae participate in the endocytosis of TNF-α in CECs and mediate its entry into the brain across BBB (Pan et al., 2007). What’s worse is that CAV-1 can also interact with TJs/AJs, affecting BBB permeability (Marchiando et al., 2010; Liu et al., 2012). However, the impact of caveolae/CAV-1 on BBB and neural function has been found diametrically opposite. *CAV-1* knockout mice exhibit AJs degradation (Siddiqui et al., 2011), increased expression of MMPs (Gu et al., 2012), BBB leakage and aggravated brain injury (Jasmin et al., 2007; Choi et al., 2016a). The overexpression of CAV-1 can prevent the degradation of TJs, exerting a neuroprotective effect (Choi et al., 2016b). One reason for the controversial results might be attributed to CAV-1 locating both in neural cells and CECs. CAV-1 in CECs has the function of inhibiting the activity of eNOS by binding to it, while the role of CAV-1 locating in neural cells has not been studied solely. Although CAV-1 has been proposed as a target for stroke or dementia (Xu L. et al., 2015), it should still needs careful consideration when applying this suggestion.

In addition to the increase in caveolae, the functional alteration of the transporters in CECs is also implicated in impaired transcellular transport. Oxidative stress is a major cause of transporter dysfunction. The CEC mitochondria are attacked directly by ROS and ATP production reduced, resulting in the dysfunction of the ATP-dependent ion channels and transport proteins. The functions of sodium-potassium pump (Na^+^-K^+^-ATPase), calcium pump (Ca^2+^-ATPase), and ABC transporter (Zlokovic, 2011) in the membrane of CECs can be altered, leading to dysregulation of intracellular calcium homeostasis, reduced activity of eNOS (Busse and Mulsch, 1990), and BBB impairment (De Bock et al., 2013). Furthermore, due to ion channel dysfunction, alteration of the ion gradients in and out of CECs and brain parenchyma affects the depolarization of neuronal membranes, impairs the neuronal and synaptic functions, and could finally lead to neuronal death (Erecinska and Silver, 2001). In individuals with AD and asymptomatic individuals with high risk of dementia, the glucose uptake rate has been shown to be reduced in CECs (Herholz, 2010); one possible explanation is the function of GLUT-1 is impaired, which has been demonstrated in patients with AD (Mooradian et al., 1997). Therefore, the proper functioning of transporters is essential for the preservation of cognition.

#### Neurovascular Uncoupling

In cerebral hypoperfusion, the NVC is disrupted manifesting in two aspects: CBF dysregulation and the loss of trophic effect of CECs to neural cells. Since endothelium-derived NO plays a vital role in CBF regulation, CEC dysfunction diminishes the production of NO, which could lead to a neurovascular uncoupling, but controversial ideas exist. One study performed in mice with genetic depletion of *eNOS* shows that the functional hyperemia was reduced in the somatosensory cortex evoked by whisker stimulation and by administration of Adenosine triphosphate (ATP) (Toth et al., 2015), indicating the role of eNOS in functional hyperemia is essential. However, it was also found that the role of eNOS is not essential in functional hyperemia by comparing the CBF increase in *eNOS* mutant and wild type mice (Ayata et al., 1996). Even more confusingly, another study has found eNOS is a mediator of the nitration effect of ANGII and thus mediates the adverse effect of ANGII on functional hyperemia (Girouard et al., 2007). As we discussed in NVC part, the NO mediating functional hyperemia may be mainly from nNOS, and the degree of the effect of eNOS-derived NO still needs to be future studied.

With respect to NVTC, growth factors such as BDNF secreted by CECs are downregulated in oxidative stress, leading to the decreased neurotrophic function of CECs. *In vitro* experiments have shown that cerebral endothelial oxidative stress reduces the expression of BDNF via β1-integrin/integrin-linked kinases (ILK) signaling in CECs, while the use of antioxidant increases BDNF (Guo et al., 2008). The increased TNF-α in the brain of the elderly and patients with hypertension and hyperlipidemia could also reduce BDNF expression by binding to TNF receptor 1 (TNFR-1) in CECs (Xu H. et al., 2015). Since the normal BDNF expression is eNOS-dependent, BNDF function is also impaired when eNOS is dysfunctional. The decreased BDNF level leads to impaired angiogenesis, neuronal survival, as well as, synaptic plasticity, whereby cognitive impairment occurs (Marie et al., 2018).

#### A Vicious Circle of Cerebral Hypoperfusion, CEC Dysfunction and CBF Dysregulation

The CEC pathological and functional changes are discussed above under the state of cerebral hypoperfusion. Actually, cerebral hypoperfusion also impairs other vascular parts (e.g., VSMCs and pericytes) which worsens CBF dysregulation, and further impairs CEC function.

With the risk factors such as aging, obesity, and hypertension which always coexist with cerebral hypoperfusion, vascular structure is vulnerable to be compromised. Stiffness of cerebral arterial wall, reduced vessel diameter, and decreased vascular elasticity diminishes CBF and aggravates hypoperfusion further (Baumbach et al., 2002; Hamel, 2006; Pires et al., 2013). As one of the types of VaD, cerebral autosomal dominant arteriopathy with subcortical infarcts and leukoencephalopathy (CADASIL) manifests intrinsic remodeling of the cerebral vessels due to Notch-3 mutations, accompanied by a gradual decrease in capillary density and a decrease in CBF (Joutel et al., 2010; Joutel and Faraci, 2014; Dabertrand et al., 2015). Therefore, the mutual interaction of vascular structural changes and hypoperfusion also represent an important contributor to cognitive impairment.

Pericytes always are early responders in cerebral hypoxia. Aging reduces coverage rate of pericytes around CECs (Bell et al., 2010; Tucsek et al., 2014); pericyte loss and degeneration are also found in animal models of hypertension (Suzuki et al., 2003), hyperhomocysteinemia (Kim et al., 2002), diabetes (Price et al., 2012), and CADASIL (Ghosh et al., 2015). Given the role of pericytes in regulating TJs and functional hyperemia, pericyte alterations affecting BBB permeability and NVC also play a role in cognitive impairment (Winkler et al., 2014).

Cerebrovascular endothelial cells are the major source of vasoactive substances. In addition to endothelial NO, release of other vasoactive substances are also susceptible to cerebral hypoperfusion. For example, prostaglandin, an endothelium-derived vasodilator, reduces with age, thereby impairing vasodilation (Gano et al., 2014). Under the action of ANGII, the effect of endothelin-1 (ET-1) and thromboxane A-2 (TXA-2) are enhanced, making the hypoperfusion worse.

In general, angiogenesis is triggered as an endogenous compensation mechanism when nutrition and energy supply do not meet demand. In the elderly, attenuated angiogenesis due to impaired secretion of VEGF and decreased amount of endothelial progenitor cells (EPCs) (Lahteenvuo and Rosenzweig, 2012; Marin et al., 2013) is one of the main manifestations of vascular aging. In addition, MMPs play an important role in angiogenesis. Aging endothelial cells express high levels of TIMP-2, a natural inhibitor of MMP-9, which prevents MMP-9 from functioning in angiogenesis. The secretion of various trophic factors from pericytes is also impaired (Bell et al., 2010), influencing the renewal of endothelial cells.

Structural alteration of cerebral vasculature, pericyte impairment and reduced angiogenesis results in CBF dysregulation, continuous cerebral hypoperfusion, and persistent damage to CECs, ultimately damaging neurological function.

## Interaction Between CECs and Neural Cells — Onset of VaD

Disturbed interactions between CECs and neural cells due to CEC damage result in astrogliosis, microglial activation, white matter (WM) damage, impaired synaptic plasticity, and neural inflammation and apoptosis, on a gross level leading to cognitive impairment. Below, we speculate how CEC dysfunction affects neural cells in chronic hypoperfusion, and eventually causes VaD.

### Astrogliosis

Astrocytes are the most abundant cells in the brain, accounting for about 50% of the total cell number in the CNS (Filous and Silver, 2016). As astrocytes are located between the CECs and neurons, they are considered to play a key role in the process of neuronal damage caused by vascular factors.

Cerebrovascular endothelial cell dysfunction activates astrocytes through multiple pathways. VEGF secreted by CECs in inflammation can bind to VEGFR-1 in astrocytes and might promote proliferation of astrocytes and expression of the glial fibrillary acidic protein (GFAP) through MAPK/ERK and PI3K signaling pathways (Mani et al., 2005). Interestingly, activated astrocytes are the main source of VEGF in the brain (Acker et al., 2001). Fibrinogen deposition only occurs in the condition of junctional protein disruption in CECs and is found in elderly and patients with brain small vessel disease, exhibiting a significant positive correlation with an increased risk of dementia (Bridges et al., 2014; Chen A. et al., 2016; Hainsworth et al., 2017). Fibrinogen in the brain may be an early signal to activate astrocytes after dysfunction of CECs. It may serve as a carrier of TGF-β and promote the phosphorylation of mothers against decapentaplegic homolog 2 (Smad2) in astrocytes via the TGF-β/Smad2 signaling pathway, leading to the deposition of chondroitin sulfate proteoglycans (CSPG) and formation of glial scars (Schachtrup et al., 2010). Due to TJ dysfunction (Schreibelt et al., 2007), leukocytes infiltrate in the brain parenchyma and release inflammatory cytokines such as TNF-α and IL-1β that may trigger astrogliosis (Cui et al., 2011). In transgenic mice, the over-expression of ET-1 by CECs is a critical factor in astrogliosis and relates to the severity of cognitive impairment, (Zhang et al., 2013b); the potential mechanism is c-Jun expression activated by JNK and p38MAPK after ET-1 binding to the endothelin receptor type B (ETB) in astrocytes. Silencing c-Jun or antagonizing ETB can block this effect of ET-1 (Gadea et al., 2008). Under inflammatory conditions, however, astrocytes may express ET-1 and promote astrogliosis through an autocrine mechanism (Hammond et al., 2014).

Although astrogliosis is a protective mechanism, the over-activated astrocytes release a variety of neurotoxic substances and form glial scars, causing severe damage to neural cells and BBB integrity, which can ultimately affect cognitive function. Astrogliosis and astrocyte endfeet swelling have been found in the brains of VaD animal models (Price et al., 2018). In the development of frontotemporal dementia in humans, an increase in the number of astrocytes is the earliest anatomical change (Kersaitis et al., 2004). Proliferative astrocytes have also been found in the model of CADASIL (Brennan-Krohn et al., 2010).

Although studies have found that astrocytes co-cultured with endothelial cells can improve the function of TJs between endothelial cells under condition of hypoxia (Fischer et al., 2000; Song et al., 2002; Al Ahmad et al., 2009, 2011), the excessive activation of glial cells is a major cause of further damage to BBB in VaD. Hypoxia-induced activation of HIF-1α in astrocytes and the corresponding elevated expression of MCP-1, MMP-13, VEGF, and VEGFR-1 can lead to increased permeability of BBB (Engelhardt et al., 2014). Interestingly, MCP-1 was found to induce the redistribution of TJs via the Rho signaling pathway, resulting in increased paracellular permeability of CECs (Stamatovic et al., 2003). MMP-13 released by astrocytes under hypoxia can also lead to the impairment of ZO-1 and VE-cadherin (Lu D.Y. et al., 2009a). After brain injury, VEGF released by astrocytes binding to VEGFR-1 seems to cause BBB dysfunction via the PI3K/Akt signaling pathway (Vogel et al., 2007). In animal experiments, VEGF binds to VEGFR-2 in CECs and activates phospholipase Cγ (PLCγ)/eNOS pathway in CECs, mediating the downregulation of claudin-5 expression, exerting a critical role in abolishing BBB integrity (Argaw et al., 2012).

### Microglial Activation

Microglia are permanent immune cells in the brain, accounting for approximately 16% of the total number of CNS cells (Norden and Godbout, 2013). Microglial activation has been shown to reduce synaptic plasticity and directly affect the long-time potentiation (LTP) of the brain, which will lead to cognitive impairment (Norden and Godbout, 2013).

When CNS tissue is damaged, microglia is rapidly activated. Activated microglia displays an anti-inflammatory phenotype (M2 type) or a pro-inflammatory phenotype (M1 type). The M2 type microglia secrete protective cytokines that relieve the immune response and digest debris of necrotic tissue, promoting survival and repair of neurons (Huber et al., 2006), while excessive M1 microglia activation primarily contributes to the inflammatory environment in the brain, not only causing further damage to the BBB but also resulting in neuronal and oligodendrocyte (OL) injury. As such, microglia activation during hypoperfusion has been shown to be closely related to WM damage and cognitive dysfunction in VaD (Shibata et al., 2004; Coltman et al., 2011).

Chronic hypoperfusion causes CEC dysfunction, leading to the entry of fibrinogen and inflammatory cells into the brain tissue via the disrupted BBB that sustains the M1 type of microglia activation (Davalos et al., 2012). Studies have demonstrated that fibrinogen that enters the brain parenchyma can bind with integrin cluster of differentiation 11b (CD11b)/CD18 on microglia promoting their activation and assembly at the site of vascular injury (Adams et al., 2007; Ryu and McLarnon, 2009; Davalos et al., 2012). *In vitro* experiments have shown that MMP-3 secreted by CECs can also activate the microglia in the condition of oxygen and sugar deprivation. Furthermore, MMP-3 deficient glial cells can significantly reduce the activation of microglia (Lee J.Y. et al., 2015).

Activation of M1 type microglia can strongly impair BBB integrity. In the condition of hypoxia, sensitive microglial cells can activate HIF-1α. This leads to the expression of IL-1β, TNF-α, IL-16, and MCP-1 that can promote rearrangement of occludin, claudin-5, and ZO-1 and increase the expression of adhesion molecules in CECs, making the BBB damage rather prominent (Bolton et al., 1998; Hallenbeck, 2002; Yamagata et al., 2004; Jiao et al., 2011; Obermeier et al., 2013). Activated microglia is the main source of MMPs and ROS in the brain. MMP-2/-3/-9 are abundantly expressed by microglia and can degrade BM and TJs, leading to the disruption of BBB (Truettner et al., 2005). ROS released by activated microglia can cause the redistribution of TJs via RhoA/PI3K/Akt pathway and affect BBB function as described above.

### WM Lesion

The cerebral cortex is a high-level neural center in humans, which is closely connected to cognitive function. However, the contribution of WM in cognitive function is increasingly valued. WM occupies about half of the brain volume (Filley and Fields, 2016) and is sensitive to changes in CBF, prone to be compromised in hypoperfusion. WM hyperintensities (WMH) were discovered on the T2-weighted images of magnetic resonance imaging in elderly (Babikian and Ropper, 1987) and were proved to be related to the occurrence of cognitive impairment (Rosenberg et al., 2016). As noted already earlier, multiple VaD risk factors lead to endothelial dysfunction and CBF dysregulation, increasing the sensitivity of WM to lesions (Matsushita et al., 1994; Delles et al., 2004; Jennings et al., 2005). Gradual cerebral hypoperfusion in spontaneously hypertensive rats has been shown to result in slowly evolving WM abnormalities and impaired working memory (Kitamura et al., 2016). Bilateral common carotid artery stenosis (BCAS) in mice for 2 weeks mimicking cerebral hypoperfusion results in rarefaction and vacuolation in WM (Shibata et al., 2004). After 1 month, diffuse damage to myelinated axons in WM was detected (Holland et al., 2011; Reimer et al., 2011). Consecutively, we will delineate the loss of OL and demyelination, the two main characters of WM lesion (Wharton et al., 2015).

#### OL Damage and Demyelination

Mature OL wrap around axons to form phospholipid-rich segmental myelin that not only promotes rapid conduction of nerve impulses (Baumann and Pham-Dinh, 2001) but also provides nutrient support for axons by secreting multiple nutrients, thereby promoting axonal growth and survival (Wilkins et al., 2003). OL injury can cause demyelination and axonal degeneration. Chronic cerebral hypoperfusion has been found to exert a significant impact on the integrity of the myelin sheath, eventually causing cognitive dysfunction (Venkat et al., 2017). The density of myelin sheath and the number of oligodendrocyte precursor cells (OPCs) in WM in patients with VaD is significantly reduced (Wolf et al., 2017). Some myelin proteins have also been found to be significantly altered in VaD patients or animal models. For example, the expression of myelin basic protein (MBP), a major membrane protein, and myelin-associated protein (MAG), both highly sensitive to ischemia and hypoxia, is significantly reduced in the brain of VaD patients (Barker et al., 2013; Wolf et al., 2017). Conversely, the expression of proteolipid protein-1 (PLP-1), another myelin protein, increases with the severity of VaD (Ihara et al., 2010; Barker et al., 2013), while the proportion of MAG: PLP-1 decreases dramatically (Thomas et al., 2015).

Studies have found that after TJ disruption, TNF-α and fibrinogen entering the brain tissue can cause demyelination. In the demyelinated area, the fibrinogen, proliferating astrocytes, and activated microglia (Simpson et al., 2007) coexist, suggesting that fibrinogen and the resulting activation of glial cells may be the main cause of OL dysfunction (Marik et al., 2007). Under hypoxic conditions, OL can preferentially use lactic acid as its energy source. OL expresses a large amount of lactic acid transporter, monocarboxylate transporter-1 (MCT-1) (Lee et al., 2012), which can obtain lactic acid from astrocytes. A decreased lactate production from the astrocytes in hypoperfusion combined with the decreased MCT-1 activity in the elderly, leads to a decrease in the supply of lactic acid to OL, leading to OL damage or apoptosis. With an impaired BBB, P2X purinoceptor 7 (P2X7) in OL can bind to ATP released by neurons and astrocytes, thereby leading to increased calcium influx and consecutive damage in the OL (Domercq et al., 2010).

Cerebrovascular endothelial cell dysfunction directly leads to astrogliosis, which can indirectly cause OL damage. Based on the ability of astrocytes to uptake glutamate and astrocytes in WM expressing high levels of the glutamate-aspartate transporter (GLAST/EAAT-1) and glutamate transporter-1 (GLT-1/EAAT-2) (Goursaud et al., 2009), it is speculated that astrocytes may protect neurons and OL by reducing the glutamate accumulation within interstitial spaces. The upregulation of Na^+^-dependent GLT-1 found in aging brain (Liu et al., 2017) may serve as a protective mechanism to clear excess glutamate rapidly. However, some studies demonstrated that in hypoxia, reduced ATP supply leads to incomplete deactivation of Na^+^ channels and dysfunction of Na^+^/Ca^2+^ exchangers and elevates intracellular Na^+^ and Ca^2+^ concentrations (Stys et al., 1992), resulting in the reversal of GLT-1 function, which releases an enormous amount of glutamate (Baltan, 2009). Excess glutamate overactivates α-amino-3-hydroxy-5-methyl-4-isoxazole propionic acid/kainic acid (AMPA/KA) type glutamate receptors (Tekkok and Goldberg, 2001; Baltan et al., 2008; Fu et al., 2009) that can stimulate the accumulation of Zn^+^ in OL and activate the ERK-1/2 signal transduction pathway in a poly ADP ribose polymerase-1 (PARP-1)-dependent manner (Domercq et al., 2013), eventually leading to OL death. In addition, a study has found that MMP-2 can degrade MBP, which might be one of the possible causes of demyelination (Walker and Rosenberg, 2010). The activation of the NF-κB pathway presents a robust damaging effect on OL, and the *IκB2* knockout can significantly reduce the loss of OL (Raasch et al., 2011). OL comprises abundant iron ions and is extremely sensitive to ROS (Lampron et al., 2015). Thus, it can be speculated that OL may be sensitive to glial activation, which could be the leading cause of demyelination.

Demyelination leads to the decelerated conduction of nerve impulses, and the path of memory and cognition formation is blocked, resulting in the occurrence of cognitive dysfunction.

#### Impaired Remyelination

Myelin regeneration is mainly effectuated by OPCs (Wood and Bunge, 1991). Under physiological conditions, only a small number of OPCs occur in the brain. With tissue injury, OPCs proliferate and differentiate into mature OLs and migrate to the vicinity of axons, attempting to form new myelin sheaths. Cytokines, chemokines, and growth factors decrease the remyelination ability of OPCs (Tsai et al., 2002; Murtie et al., 2005; Aguirre et al., 2007; Arai and Lo, 2010; Zhang et al., 2010; Aparicio et al., 2013; Ferent et al., 2013; Ortega et al., 2013).

First, CEC dysfunction and the resulting cerebral inflammatory environment and oxidative stress reduce the migration and proliferative capacity of OPCs (Arai and Lo, 2009). Consecutively, oxidative stress can reduce the expression of genes that promote the differentiation of OPCs, such as *sonic hedgehog* (*Shh)* and transcription factor *Sox10*, and thus, prevent the differentiation of OPCs (French et al., 2009). CEC-derived ROS and microglia-derived ROS reduce the level of GSH and increase the free radicals in OPCs, thereby impairing the ability of OPCs to differentiate into mature OLs (Back et al., 1998). When fibrinogen enters brain tissue, it activates the bone morphogenetic protein (BMP) signaling pathway and promotes the phosphorylation of Smad-1/5/8 in OPCs that acts to inhibit the differentiation of OPCs, thereby inhibiting remyelination (Petersen et al., 2017).

Secondly, with excessive microglial activation, proliferation of OPCs is obviously inhibited, as shown by different authors (Pang et al., 2010; Taylor et al., 2010; Falahati et al., 2013; Smith et al., 2015). In a co-culture of microglia and OPCs, apoptosis of OPCs could be induced by activated microglia through HSP60/TLR4/NF-κB signaling (Li et al., 2017). Although a study has shown that M2-type of microglial activation can release activin-A to promote the differentiation of OPCs and remyelination, and M2-type depletion and blockade of activin-A inhibits myelin regenerative capacity of OPCs (Miron et al., 2013), indicating M2-type of microglial activation may be beneficial to OPC differentiation, the evidence is still weak. However, a study suggests that the non-activated microglia is indeed beneficial to OPCs (Nicholas et al., 2001). The protective role of activin-A for demyelination is in consensus, suggesting it could be a potential therapeutic target (Park et al., 2016; Stayte et al., 2017; De Berdt et al., 2018). Shredding myelin debris can interfere with the differentiation of OPCs; however, the ability of microglia/macrophages to phagocytize the myelin fragments is reduced due to advanced age and inflammatory stimuli. In addition, the persistence of myelin fragments results in the inability of OPCs to differentiate into mature OL (Miron et al., 2013).

Lastly, although the trophic and supportive role of astrocytes to OPCs has been reported (Corley et al., 2001; Arai and Lo, 2010; Barnett and Linington, 2013), the activation of astrocytes have been proven to exert a negative effect on the proliferation and remyelination of OPCs (Cambron et al., 2012; Cargill et al., 2012; Tripathi et al., 2017). CSPGs are highly expressed by reactive astrocytes and are potent contributors to hindered remyelination (Galtrey and Fawcett, 2007). In addition to the inflammatory cytokines secreted by astrocytes mediating the damage to OPCs, hyaluronan (HA), released by activated astrocytes, has been shown to accumulate in the lesion area and inhibits OPC maturation (Back et al., 2005). HA can be degraded by the hyaluronidase 20 (PH20) in the injured state, and the cleavage products can inhibit the differentiation and maturation of OPCs, thereby hindering the regeneration of myelin sheaths (Preston et al., 2013).

Interestingly, although OPCs can promote the function of TJs in CECs by TGF-β signaling, injured OPCs can express MMP-9, thereby acquiring the ability to degrade BBB (Seo et al., 2013), leading to its further dysfunction. A previous study has found that compared to the normal WM, TJ lesions are independently associated with WMH severity. Moreover, BBB integrity in the WMH region is significantly reduced (Young et al., 2008), which in turn can aggravate the WM damage.

### Neuron Injury

#### Axon Injury

Axons play a critical role in neuronal signal propagation. The myelin sheath, neuronal mitochondrial function, and energy supply affect the axonal function. Demyelination not only weakens the propagation speed of nerve impulses but also weakens or even interrupts the trophic support for axons, resulting in axonal degeneration. Moreover, the decrease in axonal protein expression occurs early in the course of hypoperfusion (Reimer et al., 2011). VaD model rats showed significant myelin loss and axonal injury (Venkat et al., 2017).

The accumulation of fibrinogen around the blood vessels, consistent with the region of axonal damage, is speculated to be an early cause of axonal injury (Davalos et al., 2012). Myelin sheaths can provide lactic acid to axons via EAAT 1/2 under normal or hypoxic conditions, serving as the main source of energy for maintaining the normal physiological function of axons (Rinholm and Bergersen, 2014). Demyelination disrupts axon lactate supply. OL-derived insulin-like growth factor 1(IGF-1) and glial-derived neurotrophic factor (GDNF) capable of increasing the axonal length and promoting neuron survival (Wilkins et al., 2003) is weakened due to demyelination. In addition, after myelin loss, axons are directly exposed to the inflammatory environment of the brain parenchyma, affecting the energy synthesis of axons, thereby altering the activity NA^+^-K^+^-ATPase, causing Na^+^ and Ca^2+^ overload and axonal flow disturbances (Stys et al., 1992; Franklin and Ffrench-Constant, 2008; Matute and Ransom, 2012).

Although demyelination is the primary cause of axonal degeneration, axonal damage may also be unrelated to demyelination. Astrocytes are in direct contact with CECs and can preferentially uptake glucose through GLUT-1, which is then converted into lactic acid to provide energy for other types of neural cells (Walz and Mukerji, 1988). Both *in vitro* and *in vivo* experiments confirmed that astrocytes could directly supply lactic acid to the axons via MCT-1/2/4 and maintain the normal physiological function under slightly hypoxic conditions (Pellerin et al., 1998; Berthet et al., 2009; Suzuki et al., 2011). GLUT-1 and MCT-1/2/4 dysfunction in CECs and astrocytes during hypoperfusion may lead to interrupted lactic acid supply to axons, causing axonal damage or degeneration. Lactic acid participates in the maintenance of LTP and is essential for the formation of memory (Suzuki et al., 2011). In this case, interrupted lactic acid supply could be a putative cause of cognitive impairment in VaD. In addition, the presence of abundant mitochondria makes axon the main source of ROS after brain injury, and thus, a primary target of oxidative stress (Lipton, 1999). Age-related and ROS-induced mitochondrial dysfunction and reduction in endogenous antioxidants increase the likelihood of mitochondrial oxidative stress leading to axonal injury.

#### Damage to Synaptic Plasticity

Synaptic plasticity refers to the characteristic of synapses that their number, morphology, and function of synapses can undergo long-lasting changes, and is considered as the neurobiological basis for cognitive function and memory storage. LTP is a typical type of enhanced synaptic plasticity and the leading molecular mechanism underlying long-term memory. Changes in the expression of synaptic-associated proteins are found to occur early in the onset of AD in patients or animal models. Although related data are limited on VaD and changes in synaptic plasticity are not as pronounced as that in AD, existing data confirm impaired LTP and increased silent synapses (Wang et al., 2016) in VaD patients and animal models (Zhang et al., 2015; Yang J. et al., 2017). These modifications are accompanied by the alterations of synaptic proteins such as synaptophysin (SYN), synaptosome-associated protein 25 (SNAP-25) (Sinclair et al., 2015; Volgyi et al., 2018), postsynaptic density protein 95 (PSD-95), microtubule-associated protein-2 (MAP-2), and growth-associated protein (GAP-43), which has been confirmed experimentally (VanGuilder et al., 2011; Yang J. et al., 2017).

Cerebrovascular endothelial cell dysfunction can directly affect synaptic plasticity. A large number of studies on *eNOS* knockout animals have confirmed that eNOS-derived NO is a key signaling molecule that maintains the synaptic function in the cortex and hippocampus. After *eNOS* knockout, the formation of LTP is rather challenging (Haul et al., 1999; Doreulee et al., 2003; Hopper and Garthwaite, 2006). Although the localization of eNOS in CNS has been controversial, studies have found that in the hippocampal area, eNOS is only expressed in CECs (Blackshaw et al., 2003). Therefore, the changes of NO signaling in CECs may be one of the early reasons leading to the impairment of synaptic plasticity. The decreased expression of BDNF due to disrupted eNOS/NO activity may as well be a cause of impaired synaptic plasticity.

Microglial activation and astrocyte proliferation induced by CEC dysfunction can directly affect synaptic plasticity. The activated microglial cells express iNOS abundantly and the released NO far exceeds the physiological requirements, which in turn, inhibit LTP formation (Wang and Han, 2018). After cerebral ischemia, blocking iNOS can restore LTP and improve behavioral performance (Mori et al., 2001).

#### Neuronal Apoptosis

Chronic hypoperfusion can lead to neuronal apoptosis, which is directly related to the CEC dysfunction. The barrier function of CECs is impaired and fibrinogen, immunoglobulin-G (IgG) and leukocytes enter the brain parenchyma, causing neuronal apoptosis. *S*-nitrosylated fibrinogen by excess NO (Jenkins et al., 2018) has been found to be able to induce the expression of apoptotic protein cysteine-aspartic proteases-3 (caspase-3) in neurons, which leads to neuronal apoptosis (Ill-Raga et al., 2015). Infiltrated leukocytes can release a variety of toxic proteins, causing neuronal injury and death. Neurotoxic granzyme-B (Gra-B), released from infiltrated lymphocytes under hypoxic conditions, binds to PARP and caspase-3, leading to neuronal death (Chaitanya et al., 2010). In addition, Gra-B activates the protease-activated receptor-1 (PAR1)/PLCβ-IP3 signaling pathway, causing neuronal death, which is further enhanced by IL-1β (Lee et al., 2017). MMP-9 expressed by CEC also plays a role in neuronal death. Although it is not yet clear that whether MMP-9 secreted by CEC can enter the brain across BBB, *in vitro* experiments found that in ischemic and hypoxic conditions, the co-incubation of neurons and CEC expressing MMP-9 can lead to neuronal apoptosis (Cheon et al., 2016). The underlying mechanism may be related to the activation of apoptosis signal-regulating kinase 1 (ASK-1) [ASK has been shown to be closely related to cognitive impairment caused by chronic hypoperfusion (Toyama et al., 2014)], inhibition of PI3K/Akt/Nrf2/Heme Oxygenase-1 (HO-1) signaling, and the upregulation of COX-2; all these may eventually lead to neuronal apoptosis (Cheon et al., 2016). Furthermore, the damage to neuronal DNA caused by MMPs has been suggested as an early event of ischemia and hypoxia (Kimura-Ohba and Yang, 2016). Although the primary targets of MMPs are extracellular matrix and TJs, under inflammatory conditions, the expression of active MMPs in the neuronal nucleus will also increase, causing neuronal DNA damage and impaired DNA repair ability by cutting PARP-1 that eventually leads to neuronal apoptosis (Kimura-Ohba and Yang, 2016).

In addition, CEC dysfunction causes activation of microglia, making that a major source of TNF-α, IL-1β, ROS, excess NO, and various chemokines, which also represent the main cause of neuronal death. Both TNF-α and IL-1β can promote the expression of intracellular and extracellular glutaminase 1 and cause the accumulation of intracellular and extracellular glutamate that leads to neuronal death (Ye et al., 2013). Moreover, TNF-α also inhibits the function of astrocyte GLT-1 by activating the NF-κB pathway, resulting in extracellular excess of excitatory glutamate. The accumulation of elevated TNF-α in the brain of the elderly may initiate phagocytosis of stressed neurons via vitronectin receptor (VNR), resulting in neuronal loss (Neniskyte et al., 2014).

Activated astrocytes can also cause neuronal damage or death by releasing inflammatory mediators. The activation of astrocytes may promote hippocampal neuronal apoptosis by over-activation of secreted lipocalin-2 (LCN2) (Kim et al., 2017).

#### Impaired Neuronal Regeneration

It is commonly accepted that glial scars formed by the excessive proliferation of astrocytes impede axonal growth (Ohtake and Li, 2015). As mentioned above, CSPGs may play a major role (Galtrey and Fawcett, 2007). However, it is suggested that the astrocyte scar is helpful for axon regeneration (Anderson et al., 2016). On the other hand, OL-expressed axon inhibitor proteins can inhibit the axon regeneration post-injury, thereby resulting in the continuous loss of axons (Xie and Zheng, 2008). Under physiological conditions, MAG, and OL-myelin glycoproteins (OMgp) expressed by OL, actin, and Nogo-A, respectively, can act on the Nogo66 receptor (NgR) and inhibit the axonal overgrowth via the RhoA/Rho-associated protein kinase (ROCK) pathway (McKerracher and Winton, 2002). However, studies have shown that hypoxia and ischemia can lead to the overexpression of NgR, which activates the RhoA/ROCK signaling pathway and inhibits the axon regeneration (Chang et al., 2016).

In the case of cerebral ischemia and hypoxia, NSCs in adult brain subventricular zone (SVZ) can migrate to the neuronal injury area, initiating neural regeneration. The migration and differentiation of NSCs are affected by their regenerative ability. BDNF derived from CECs is a crucial factor that promotes NSC migration. The oxidative stress and inflammation lead to decreased BDNF and impaired neural regeneration (Guo et al., 2008). Neurite growth inhibition can also be mediated by fibrinogen. Fibrinogen that enters the brain binds to neuronal β3 integrin and induces epidermal growth factor receptor (EGFR) phosphorylation in neurons, thereby inhibiting neurite outgrowth (Schachtrup et al., 2007). Both CECs and microglia produce a large amount of NO, which impairs neuronal regeneration. Several studies have found that a large amount of NO can promote the differentiation of NSCs into astrocytes and reduce the occurrence of neurons, thereby impairing neuronal regeneration post-injury (Covacu et al., 2006).

## Conclusion

**FIGURE 2:**
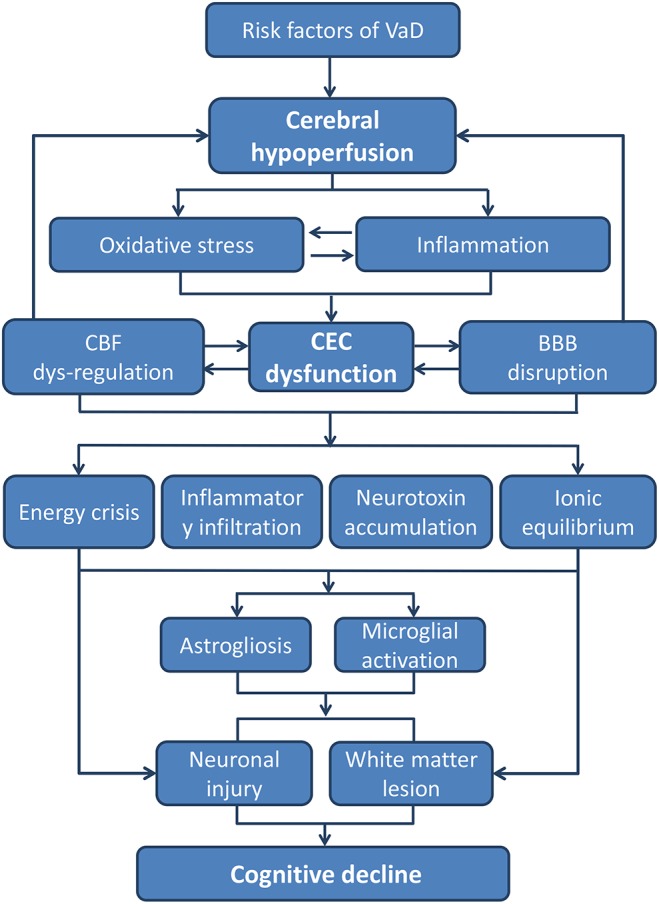
The pathways from CEC dysfunction to cognitive decline. The core pathological characteristics of CEC dysfunction are oxidative stress and inflammatory response, which could be the result of hypoperfusion due to all kinds of VaD risk factors. The two aspects of CEC dysfunction can both lead to a neurotoxic environment for neurons and white matters, whereby cognitive decline and dementia occurs eventually (BBB, blood–brain barrier; CEC, cerebrovascular endothelial cell; CBF, cerebral blood flow; VaD, vascular dementia).

In summary, VaD occurs due to advanced age and multiple vascular risk factors. Existing or secondary triggered CEC dysfunction can be regarded as basic pathological aspect of VaD. CEC dysfunction is mainly related to exposure to oxidative stress and inflammatory environment caused by factors such as hypoperfusion, advanced age, and cardiovascular diseases. Under the influence of inflammation and oxidative stress, glial cells in the CNS are activated and finally lead to WM lesion and neuron damage (Figure 2). As summarized in our review, the impairment of the eNOS/NO signaling pathway seems to play a major role in CEC dysfunction and subsequent neural injury. Therefore, targeting the eNOS/NO signaling pathway in CECs not only protects from CEC dysfunction but as well indirectly promotes neuroprotection. On the other hand, a weakened barrier function of CECs is a main reason for the decline of cognitive function. In this process, the activation of the RhoA signaling pathway increases the paracellular permeability and promotes the entry of leukocytes and plasma proteins into the brain and is one of the major causes of glial activation and neuronal damage. Although the RhoA signaling pathway has been widely studied in cardiovascular diseases, it is rarely investigated with respect to CNS diseases, especially VaD. CAV-1 plays a critical role in transcellular permeability of CECs and may also serve as a target for future drugs for treating VaD. The beneficial effects of the alteration of CAV-1 in the brain are still controversial and currently under intensive research. However, the expression of CAV-1 in CECs is not sufficiently explored. Drugs that regulate the expression of CAV-1 in CECs may function without crossing the BBB, which is of great significance for bridging the BBB bottleneck in the development of CNS drugs.

In addition, since CEC dysfunction preludes the pathogenesis of VaD, measures preventing CEC dysfunction in populations with high-risk factors will be beneficial in reducing the incidence of VaD. Preventive measures against diseases such as hypertension, hyperlipidemia, diabetes, and atherosclerosis are able to reduce the damage of CECs and changes in vascular structure, thereby preventing the occurrence of VaD in the elderly. For example, studies have found that stringent blood pressure control can reduce the WM damage, and a low-fat diet can also reduce the risk of dementia. Maintaining blood pressure, blood lipids, and fasting blood glucose at the target level benefits cognitive function in later years (Iadecola, 2013). On the other hand, it is foreseeable that drugs that improve the CEC function prevent the occurrence of VaD. However, such drugs still need to be developed. Several traditional Chinese medicine (TCM) preparations have been shown to reduce the production of ROS in CECs, downregulate the expression of inflammatory factors and MMPs, reduce the leukocyte recruitment and adhesion, and improve the BBB function. For example, ginseng has been widely used for the treatment of dementia, and both clinical and animal experiments have shown that ginseng has anti-inflammatory and anti-oxidant effects (Patel and Rauf, 2017). It promotes NO release in CECs, reduces leukocyte and platelet adhesion, and improves the CEC function. Resveratrol reduces the production of ROS in the brain of aged mice and downregulates the activity of NADPH oxidase, thereby restoring CEC function and NVC process (Ungvari et al., 2007, 2010; Toth et al., 2014). However, The Chinese herbal formula, rather than single compound or herb, is the most commonly used manner of treatment in TCM system. Other than the monarch herb/herbs, which is/are designed to treat the main symptoms, there are further ministerial and adjuvant herbs, which help to strengthen the effect of monarch herb/herbs or treat the accompanying symptoms. As such, there are multiple components in a Chinese herbal formula. Determining active components in a Chinese herbal formula and exploring their specific targets in the human body is challenging. Though Chinese herbal formulas also have the effect to prevent CEC dysfunction, the underlying mechanism should be energetically investigated by an integrative approach combining fingerprints, histochemistry, molecular biology, bioinformatics, and omics in conclusion Chinese herbal preparations with efficacy will be proven valuable in the prevention and treatment of VaD.

## Author Contributions

FW and LM collected the literatures. FW and YC contributed to manuscript preparation and revisions. YC drew the figures. FW, HP, and WDR contributed to manuscript revisions. HL was responsible for every step related to this manuscript, designed the study, revised the manuscript, and made the final decision to submit the manuscript for publication.

## Conflict of Interest Statement

The authors declare that the research was conducted in the absence of any commercial or financial relationships that could be construed as a potential conflict of interest.
